# The Bacterial Vaccines in Relation to the Ordinary Pyogenic Processes1Read at the Buffalo Academy of Medicine, section on Medicine, May 12, 1908.

**Published:** 1908-07

**Authors:** Norman K. MacLeod

**Affiliations:** Buffalo, N. Y.; Recently of the Immunisation and Medical Research Laboratories of the Toronto General Hospital; 327 Delaware Avenue


### The Bacterial Vaccines in Relation to the Ordinary Pyogenic Processes[1]

By NORMAN K. MacLEOD, M. D., Buffalo, N. Y.

Recently of the Immunisation and Medical Research Laboratories of the Toronto General Hospital.

I DO not propose to occupy your attention this evening either with an academic discussion of the theories of immunity or with the manner of production of the complex protective substances elaborated by the system, but rather to approach immunity from the standpoint of a practical physician or surgeon called upon to deal with diseases of bacterial origin. Hitherto the therapeutist has, in many ways, attempted to combat bacterial invasion by the use of the various antiseptics known to destroy microörganisms in vitro. Believing that it was only necessary to bring the strong antiseptics into contact with the bacteria in order to destroy or, with the weaker solutions, attenuate them, many ingenious methods have been conceived. To generalise: First, they have applied to surface bacterial affections with signal success in some of the more acute cases, but with doubtful benefit in the more chronic conditions, such as sycosis, furunculosis, carbuncles, tuberculosis and acne. Second, they have been administered per oram, either with the hope that they would destroy bacteria present in the intestinal tract or, after absorption, have an attenuating influence upon the infection. Here, however, even the most optimistic of antiseptics can point to only two conditions in which the drugs have had any control whatever over the infection; I refer to syphilis and malaria, and these are diseases of doubtful bacterial origin. Third, they have been projected into the blood stream, with the idea that they would have some effect upon the microörganisms in malignant endocarditis and also, would further influence the infective emboli set free in the blood stream. The success of this last method needs no comment.

In the early days of Listerism the fact that improvement was observed in a septic process after the application of antiseptic

14. Journal Medical Research, Vol. XVI., 1907.

1. Read at the Buffalo Academy of Medicine, section on Medicine, May 12, 1908.

dressings, or its irrigation with antibacterial fluids was taken as proof that the antiseptics were responsible for the death of the infecting organism and the return of the patient to health. The consideration that the body fluids were important factors in overcoming local or general infective processes and that the antiseptics might possibly be playing a less important role was lost sight of for the time. The observations however, that all processes were materially benefited by placing the patients under favorable hygienic conditions, by giving them suitable food and tonics, by the use of fomentations instead of cold applications, and by the inducement of localised hyperemia paved the way for our present belief, that all treatment in bacterial infections must be directed to the determination toward the infection of an adequate immunising response within the body itself.

Without further preface, then, may I suggest to you that control of bacterial invasion should be attempted by facilitating with natural stimulation nature's own method of the elimination from the human body of infecting microörganisms. I mean by this the production of immunising substances specifically directed towards the particular infective agent. May I recall to your minds the manner of control among others of enteric fever. I need hardly say that a fatal issue in typhoid is escaped by the production within the body of such immunising substances as the agglutins upon which depend the Widal agglutination test. With the increase of these substances there occurs a corresponding decrease in the severity of the symptoms of the disease. Similarly in pneumonia, by appropriate measures, it can be demonstrated that the crisis is associated with a rapid rise of the protective substances opsonins. In both of these acute conditions the mechanism of immunity has been set in motion by certain stimuli derived from the bacteria themselves and to which I shall presently refer. With respect to the majority of chronic bacterial infections such as sycosis, acne, and furunculosis, there is however but little or no protective response to the infective agent, and the manufacture of immunising substances is minimal in quantity—hence their chronicity.

I have not the space to discuss the many varieties of protective substances, such as the bactericidal substance, the bacteriolysins, the agglutins, et cetra, which bear upon the question of immunity; their actions are very complex ones, and, up to the present, having but little practical application, do not enter into the scope of this paper. I may be allowed however for continuity to briefly define the action and production of the one protective substance upon which rests the foundation of the therapy it is my purpose to discuss. I refer to the opsonins of Wright and Douglas. I need hardly say that for years, and now more than ever so, the phenomenon of phagocytosis has been credited with an important function in the control of bacterial infections, but the precise manner by which phagocytosis was brought about, has only comparatively recently been demonstrated.

Wright and Douglas in 1903, by an ingenious technic, showed that phagocytosis was impossible unless the bacteria were first acted upon by some constituent of the blood serum. To this constituent they gave the name opsonins. That is to say that the leukocytes of themselves were unable to phagocyte bacteria unless the latter had been suitably prepared by the opsonins of the blood fluids. By a further elaboration of their technic they demonstrated the feasibility of measuring comparatively the quantity of an opsonin present in any given case. This measurement they designated the opsonic index. What then does this term mean? If I were to say that the opsonic index of a patient the subject of boils was .5, I would mean that his blood contained, as compared with that of a healthy normal individual, but one-half of those substances necessary to combat the staphylococcic infection present. In addition, a study of hundreds of cases has shown that in localised affections such as boils the latter condition obtained, that is that the index was low .6 or .7; and further that with the rise of the index to 1.2 or 1.3 from appropriate therapy, the infection has rapidly subsided. This then is our object—to stimulate by some means those factors which are concerned in the natural protection of the body from bacterial invasion. And it is now proved beyond question that in the dead bodies of the specific bacteria themselves we have preëminently the most potent therapeutical agent to thus stimulate the mechanism of active immunity in certain diseases; that is to say, that where a staphylococcic infection obtains, if we inoculate the subject with an appropriate number of dead staphylococci, the body itself in response elaborates a sufficiency of antibodies or protective substances to combat the infective process present.

To continue slightly further to the better understanding of the process it might be said that consequent upon either the inoculation of a bacterial vaccine or the introduction within the body of an infective agent, the absorbed protoplasmic cell constituents of the bacteria themselves attack and combine with the receptors of certain of the body cells. If a great number of receptors are attacked and the poison is a virulent one, the whole cell may participate in the death of its receptors, but if, on the other hand, only a limited number are destroyed then the cell replaces these with receptors in excess of the original number. To such an extent may this overcompensation of the receptors reach, that the cell body is unable to retain them all attached to itself. Consequently some are cast off free into the surrounding lymph, constituting the free antibodies of active immunity. These antibodies then are the protective substances elaborated by the mechanism of immunity in response to the toxic properties of the living or dead bacteria.

I will now pass on to a brief description of the preparation of a vaccine. By the bacteriological methods usually pursued the organism, or in mixed infections the predominating organism, is isolated in pure culture and the opsonic index estimated by Wright's technic. The preparation of the vaccine is next preceded with by inoculating from 4 to 6 tubes of suitable culture-medium with the pure culture. Upon obtaining a sufficient growth the bacteria are freed from the medium by washing with normal saline; the resulting emulsion is allowed to sediment in order to remove any masses or clumps of bacteria present. After sedimentation a small sample is set aside for purposes of standardisation and the remainder sterilised by subjection to a heat of 60 degrees centigrade for one hour. At the expiration of this time, the emulsion is removed and another small portion taken and planted out to prove that devitalisation of the bacteria has been complete. Standardisation of the previously removed sample is then, by an ingenious technic, performed. Granting that the vaccine is sterile it is now ready to be diluted to any convenient and suitable strength; this is a simple matter, normal saline being the diluent to which previously has been added, as a safeguard, .25 per cent. carbolic acid.

In presenting to you the following cases I have been actuated by a desire to bring to your notice as many reports as possible, in the brief time at my disposal. I have therefore refrained from introducing any details that are not absolutely essential to the presentation of the case. Since all the conditions are those that are met with in ordinary routine practice, I have not attempted to enter into technical characteristics but merely to portray them as they appeared to us.

CONDITIONS DUE TO THE STAPHYLOCOCCUS ALBUS AND AUREUS BOILS AND CARBUNCLES.

CASE I. (B).—The patient, a man aged 49 years, had a carbuncle seven months previously; this was followed up by an uninterrupted series of boils. Upon examination the head was fixed and the whole neck hard, indurated, red and considerably swollen. Pus was expressed from several openings. The condition was of such character that the man had had to give up his employment as telegraph linesman some weeks previous to his first visit to us. His resistance was low, the index being .68. Forty-eight hours after the first inoculation the index was 1.3, the head was freely moveable, the pain and tenderness practically gone. At the end of ten days the neck was clear. There has been no recurrence. The dose of the vaccine was 100 to 200 millions of the mixed albus and aureous, the proportion of the albus to the aureus being 1 to 4.

CASE 2. (H).—The patient, a man aged 19 years, has been subject for the past ten years to periodic outbreaks of boils. The attacks have always been severe and have been accompanied by considerable pain and constitutional disturbance. The present attack, of three weeks' duration, consists of a series of boils localised to the left forearm and making their appearance in crops. Upon investigation the index was low, being .73. Inoculations were instituted and were accompanied by a rapid rise of the index. Improvement was immediate, recovery being complete in one week. The dose of the vaccine was 100 to 200 millions of the mixed albus and aureus.

CASE 3. (W).—The patient, a man aged 33 years, has had boils almost continuously for the past seven years. They have been of a severe type, accompanied by considerable pain and constitutional disturbance, and have not been localised to any particular situations. The index was low, being .81. Twenty-four hours after the first inoculation the condition was wonderfully improved and rapid recovery ensued. The dose of the vaccine was 100 to 200 millions of the mixed albus and aureus.

CASE 4. (T).—The patient, a man aged 54 years, has for one week suffered from an extremely large carbuncle in the back of the neck. At the time of the first visit the neck was extremely swollen and painful, the head was fixed. A small incision was made and the pus evacuated. His resistance was low, the index being .82. Twenty-four hours after the first inoculation all pain and tenderness had practically gone and the neck was considerably reduced in size. Recovery was complete in one week. There has been no recurrence. The dose of the vaccine was 200 millions of the mixed albus and aureus.

CASE 5. (H).—The present case is of interest in that it has been one of the few which have not quickly responded to treatment. The patient, a man aged 50 years, had a felon on the finger in November, 1906. This was followed by a carbuncle on the adjoining finger. Soon after the appearance of the latter, boils and carbuncles of severe character and accompanied by considerable sloughs, made their appearance on the back and various parts of the body. After these had shown no signs of abating he was inoculated with a vaccine. In June of last year he presented himself to us. At that time he was practically an invalid, boils and carbuncles appearing, at short intervals, all over the body. Upon inoculation improvement was slow, the boils appearing but rarely coming to a head; they were in addition much smaller than formerly, were localised to the back of the neck and were accompanied by very little pain. By the end of January, he was completely under control, the last carbuncle which made its appearance being then aborted. He has had no recurrences. The dose of the vaccine was 50 to 200 millions of the mixed albus and aureus.

CASE 6. (L).—The patient, a man aged 27 years, gave a history of boils for two months. When first seen he had a very large one on the right buttock and another beginning in the anal region. Upon investigation his index was low. Inoculations were instituted with marked and rapid relief of the larger boil; the smaller was aborted and gave little inconvenience. The condition was cured in one week. There has been no recurrence. The dose of the vaccine was 200 millions of the mixed albus and aureus.

I do not wish to make any comment upon these cases except to say that they were all of great severity and had shown no signs of improvement under other treatment prior to being referred to us. I may also add that the cases mentioned below are all of the same type as the ones just quoted and of equal severity. Of 27 cases of furunculosis treated recently in series in the laboratory twenty-one yielded rapidly to treatment, three were cured under prolonged inoculation and three were materially benefited but not cured. In the same period five cases of carbuncles of extreme severity were treated; of those three were cured rapidly, one resisted for some time and one was much improved but not cured.

SYCOSIS BARBAE.

CASE 1. (R).—The patient, a man aged 30 years, contracted a severe infection two weeks previous to August 10, 1907. The chin was first affected but the condition rapidly extended over both cheeks and down on to the neck. At his first visit to the laboratory the skin was painful, red and thickened with numerous crusts present. His resistance was low, the index being .73. Five days after the first inoculation the infiltration and pain had quite disappeared and a rapid recovery ensued. Subsequently there have been two recurrences at the old sites, but these have rapidly yielded in response to 1 to 2 inoculations. There has been no recurrence in the last six months. The dose of the vaccine was 100 to 200 millions of the mixed albus and aureus.

CASE 2. (E).—This patient, aged 30 years, and at present employed by the hospital, has had an intractable sycosis involving all of the bearded regions of the face. The condition has been present for several years, has resisted hospital treatment during that time both in England and Canada and has been of such severity that the man has not been able to retain any employment. Even in the hospital other employees have refused to share the same room with him. On examination the skin of the face was infiltrated and extremely thickened, presenting a mass of papules and pustules with the formation of large crusts. The index was low, being .63. At the end of two months' treatment the face was almost clear and at the end of five months completely so. One recurrence has since been noted, but this rapidly subsided upon a few inoculations. The dose of the vaccine was 200 millions of the mixed albus and aureus.

Case 3. (G).—This patient, a man aged 28 years, became infected prior to his departure from England. When he was transferred to us from the skin department the condition had been present three months and was of a very severe type. The whole face was a mass of pustules and crusts; in addition a bad pyorrhea alveolaris existed. The index was low, being .74. Forty-eight hours after the first inoculation the patient stated that he was decidedly better, that there was less pain and burning and that he was more comfortable. Improvement was rapid and in less than two months the face was quite clear. There has been no recurrence.

It is interesting merely as a coincidence to note that the pyorrhea alveolaris improved considerably under the vaccine. The dose of the vaccine was 100 to 200 millions of the mixed albus and aureus.

Case 4. (K).—This patient was one of the worst and most intractable cases in our experience. He had had the condition for many years and had never found any benefit from the various treatments instituted. The skin itself was in a deplorable condition, great masses of crusts covering the whole swollen surface. The index was low, being .61. Improvement was steady from the time of the first inoculation but the skin was not clear until four months treatment had been given. There have been no recurrences. The dose of the vaccine was 100 to 200 millions of the mixed albus and aureus.

In commenting upon sycosis it may be said that in it the vaccine therapy has had its most striking results. In cases pronounced hopeless after years of treatment, the improvement after inoculations has been pronounced, and, in the majority of cases, comparatively rapid. I may say that after the inoculations have been instituted all other treatment has been discontinued, except that in the beginning warm moist dressings of boracic acid have been applied to allay discomfort.

### ACNE.

Case 1. (G).—This patient, a school girl, presented herself to us for treatment in March last, suffering from acne indurata of two years' standing. She had been under continuous treatment during that period with no signs of improvement whatever. The condition was present on both sides of the nostrils. The skin of the face looked unhealthy, having a bluish tinge. Hard indurated areas were present and these from time to time were accustomed to break down and to discharge pus. Five weeks after inoculation the improvement was marked, in ten weeks the process had completely subsided and the complexion of the face was clear and healthy. The dose of the vaccine was 15 to 20 millions of the albus.

Case 2. (K).—This patient, a man, had acne of the face for years. At the time of the inspection the face was covered with spots, from many of which pus was able to be expressed. There were but few comedones to be seen. Cultures revealed an albus. Inoculations were begun and fourteen days later the face was quite clear. The dose of the vaccine was 15 to 25 millions of the albus.

Case 3. (F).—This patient has been subject to acne of the face for two years. On the face were many pustular spots and comedones to be seen; the case was a severe one. An albus was obtained and a vaccine prepared. After the third inoculation the condition had improved considerably in that there were fewer pustular spots and the skin had a more healthy appearance. Cure was complete in five weeks. The dose of the vaccine was 15 to 25 millions of the albus.

I may say that in acne the vaccines obtain some of their most striking results as well as some of their most striking failures. If an albus can be isolated from the pustular spots the prognosis is more favorable. Frequently, however, no growth can be obtained. Those cases due to the bacillus of acne present an unfavorable prognosis unless the bacillus can be obtained and a vaccine prepared. In every case the prognosis must be guarded.

CONDITIONS DUE TO THE STREPTOCOCCIC INFECTIONS.

Case 1. (A).—This patient was a well nourished man of 35 years of age. The following conditions were observed at our first examination. First, a varicose ulcer of ten months standing with a free discharge. Second, two septic incision wounds above the ulcer and consequent upon a phlebectomy performed five weeks previously for relief of the ulcer. Third, a swelling, red and indurated, one inch in diameter, situated over the external malleolus of the same leg and opened three days previously. A staphylococcus pyogenous aureus was demonstrated in the ulcer and stitch wounds. The index was .86. Upon inoculation improvement was rapid except in the swelling over the malleolus. At this time another swelling made its appearance in the back of the leg; it extended with astonishing rapidity and soon broke down. Cultures from this latter and from the malleolar process revealed a streptococcus. A vaccine was prepared. At the time of the first inoculation with the streptococcic vaccine the leg was in the following condition: first, the ulcer was almost well and had very little discharge. Second, the lower incision wound was closed. Third, the upper wound was closed except in the upper one-half inch. Fourth, the swelling over the malleolus, now two inches in diameter, red and angry looking, had a free discharge. Fifth, the whole back of the leg was hard and indurated, with numerous areas breaking down; there were also two openings present with pus discharging freely. The whole leg was extremely swollen from two inches above the knee down to and including the ankle. The pain and tenderness was extreme. Twenty-four hours after the first inoculation of the streptococcic vaccine the change was remarkable, the swelling was markedly reduced, the pain and tenderness practically gone, the patient was comfortable. At the end of eight days the leg was almost free from the streptococcic infection; the ulcer had ceased to discharge and soon healed over after the application of an Unna's dressing. The upper incision closed upon the removal of an old suture. The dose of the vaccines were 100 to 200 millions of the mixed albus and aureus, and 5 to 10 millions of his own streptococci.

CASE 2. (M).—This patient was a case of acute empyema which had been operated upon eight weeks previous to our first visit. At that time a sinus was seen with a considerable but not profuse discharge. The lung had expanded. A streptococcus was observed in the cultures and a vaccine was prepared. At the end of one week the discharge had ceased and the sinus closed. The dose of the vaccine was 10 millions of his own streptococci.

CASE 3. (C).—The patient sustained an injury to the foot three months prior to treatment. A septic condition supervened involving the plantar surface of the foot. Two operations were performed but did not stop the process. Further operative procedure was postponed for fear of destroying the arch of the foot and pending the use of the vaccines. At the time of observation the foot was swollen and a sinus discharging a considerable amount of pus was seen. A streptococcus was isolated and a vaccine prepared. Six weeks later the pain and tenderness had completely disappeared and the sinus nearly closed. The recovery was uneventful. The dose of the vaccine was 10 millions of his own streptococci.

CASE 4. (B).—This patient had been suffering from a chronic osteomyelitis of the jaw for ten weeks. Two operations had been performed but were necessarily limited on account of the proximity of the mandibular articulation. Cultures revealed a streptococcus. Upon inoculation the process subsided in nineteen days. The dose of the vaccine was 10 millions of his own streptococci.

CASE 5. (S).—This patient, a man aged 48 years, had had a severe attack of erysipelas nine weeks prior to his first visit to us. Two weeks previously a swelling appeared over the left mastoid; this soon broke down and discharged pus. At the time of visit the swelling was about two inches in diameter, strictly localised, red and painful, and with a profuse discharge; the head was fixed. Presuming the infection to be staphylococcic he was inoculated with that vaccine, but a culture was also taken. He showed no improvement and upon investigation a streptococcus was isolated from the culture and a vaccine was prepared. Twenty-four hours after the first inoculation with the latter vaccine the pain was markedly diminished, the discharge less and the head freely movable. He made a rapid recovery in one week. The dose of the vaccine was 10 millions of his own streptococci.

CASE 6. (G).—This patient, a man aged 35 years, knocked his shin against an obstruction two weeks previous to our first visit. A small abrasion resulted. This soon became sore and angry looking. At the time of the first observation the surface surrounding the abrasion was edematous and bluish black in color. There was considerable undermining present and a profuse discharge with considerable amount of sloughing to be seen. The temperature was 103° and the leg in a grave condition. Cultures were taken and a streptococcus vaccine prepared. At the time of the first inoculation the leg and thigh were extremely swollen and edematous, pus was burrowing in all directions along the muscular and fascial planes, and numerous incisions were present for its exit; an erysipelatous condition was extending rapidly over the surface of the abdomen from the thigh; numerous pyemic abscesses were present in all parts of the body; all hypodermic injections and even slight bruises were followed by the rapid formations of abscesses. The man was in a semicomatose state, the temperature being 105.5°. Subsequent to inoculation the temperature dropped to 102°, the man became rational and stated that he felt better. The redness over the abdomen ceased to spread and became less marked in color; abscesses ceased to form about the needle punctures. He was held in this condition for eight days when he gradually sank and died. The dose of the vaccine was 5 to 10 millions of his own streptococci.

CONDITIONS DUE TO THE PSEUDO-DIPHTHERIA.

CASE 1. (W).—This patient, a man aged 20 years, suffering from an empyema subsequent to influenza, came under observation four weeks after operation. Cultures demonstrated a pseudo -diphtheria bacillus and a vaccine was prepared. At the time of the first inoculation the temperature was 101°, the discharge profuse, the patient feeling ill and irritable, with no appetite. Forty-eight hours after inoculation the temperature was normal, the discharge less and the patient was feeling better in every way. The improvement was rapid and constant, the lung expanding with the decrease of the discharge. The dose of the vaccine was 50 to 100 millions of his own pseudo-diphtheria bacilli.

CASE 2. (J).—This case presented the same history as the preceding one—namely, empyema following an attack of influenza. An organism resembling the one in the previous case was isolated from an extremely mixed infection and a vaccine was prepared. The improvement in this case was correspondingly marked except that the temperature did not remain normal, rising subsequently to the first fall to between 99° and 100°. The odor of the discharge however disappeared and the same feeling of physical fitness obtained. The lung in this case did not expand as it did in the former case and the improvement was less marked. The dose of the vaccine was 100 to 200 millions of his own pseudo-diphtheria bacilli.

Before closing these cases I would like to draw your attention to the marked physical improvement of the patients. Thirty hours after inoculation they both felt considerably improved, they looked better, their appetites were good and they were more cheerful. They increased in weight to a marked extent. I would also like here to urge the dictum, that in all cases of empyema, cultures should be made at the time of the diagnostic aspiration, thus avoiding the trouble and difficulty of isolating the predominating organism from a very mixed infection such as obtained in each of these two cases.

In conclusion, it may be said that in staphylococcic infections the efficacy of the vaccines is, in our minds, beyond question. The results have been so uniformly satisfactory in boils, carbuncles, septic infections following wounds, sycosis, felons and the like, that we feel no hesitation in advocating, that we have in them the most valuable assets at our disposal today. In regard to the vaccine itself, there can be no doubt that a stock vaccine is here possible, but I would like to point out that every staphylococcic vaccine is not suited to be used as a stock vaccine. It is rarely we find one which gives uniform and satisfactory results in all cases, in addition the dosage of almost every vaccine varies considerably; it is only after repeated trials that we either adopt a vaccine or settle upon the appropriate dose. Again every case has to be considered from other standpoints than that of vaccination. The question of the isolation of the focus from the influence of the body fluids must be considered; it is manifestly impossible for the protective substances to have any influence upon a focus walled in by a thick pyogenic membrane; again, any patient who is in a pronounced anemic condition can scarcely be expected to respond to inoculation since those factors which have to do with the manufacture of the protective antibodies are evidently suffering with the general body depression. Finally, the question of the clotting power of the blood must be born in mind; frequently cases with a high clotting power do not respond satisfactorily until this has been lowered by appropriate means. In regard to the streptococcic infections we must assert that, with the exception of the streptococcus erysipelatis, a stock vaccine, in our opinion, is valueless. Polyvalent vaccines composed of strains of bacteria, that have been of the greatest benefit in their specific cases, have proved of no avail whatever when used indiscriminately.

Finally, in the employment of the bacterial vaccines problems are encountered as variable and as difficult as those met with in medicine and surgery. No set rules can be laid down to guide us, no adherence to any one line of treatment will ever be followed by successful results. In the application of this new therapy the underlying fundamental principle must not be lost sight of, it must not be forgotten that we are fighting the mischief not directly but indirectly, that the body itself is the chief factor in the fight and that if it be not in a fit condition no stimulation supplied by us will evoke a fitting response in it. And lastly the use of the bacterial vaccines or of any other immunising procedure must never be looked upon as something standing apart or alone, but rather as an adjunct to those other treatments at our command, and always as subservient to sound and practical clinical experience and observation.

327 Delaware Avenue.

